# Vaginal Celiotomy

**Published:** 1911-04

**Authors:** 


**Vaginal Celiotomy.** By **S. Wyllis Bandler,** M.D., Fellow of the American Association of Obstetricians and Gynecologists; Adjunct Professor of Diseases of Women, New York Post-Graduate Medical School and Hospital. Octavo, pp. 450, with 148 original illustrations. Philadelphia and London: W. B. Saunders Co. 1911. (Cloth, $5.00.)

It might seem that a book on vaginal celiotomy was quite superfluous, in view of the many treatises which have appeared in recent years on operative gynecology and pelvic surgery. However, Bandler has done the surgeon a great service in giving him a book which is devoted in detail to the various operative undertakings performed through the vagina. The book is beautifully illustrated and brings the subject by reason of it's splendid plates to the surgeon in a most acceptable and comprehensive manner, and the descriptive remarks attached to the illustrations delineate the various steps and stages of the operation most satisfactorily.

The author has not permitted his enthusiasm for the vaginal method of operating to run away with his judgement and experience, as gained by other methods of attack, and only in particular and specialized fields does he recommend vaginal procedures.

The chapter on cystocele and vaginal fixation is admirable, and although we do not feel satisfied with these methods of dealing with a prolapsed bladder, they nevertheless represent the views of many of the most distinguished German and continental operators. In our judgment, the mechanics of a falling bladder are not met surgically or anatomically by any operation which makes dense adhesions between the uplifted bladder and the uterus fixed forward in extreme anteflexion. The chapters on simple vaginal hysterectomy and the plates are the best we have seen on this subject.

The book is gotten up beautifully, the subject matter is nicely arranged, the type, printing and paper of the highest order, and the index is full and comprehensive in every respect. We cheerfully recommend it to the general surgeon as well as to the gynecologist.

